# A Multiomics Approach Unravels New Toxins With Possible *In Silico* Antimicrobial, Antiviral, and Antitumoral Activities in the Venom of *Acanthoscurria rondoniae*

**DOI:** 10.3389/fphar.2020.01075

**Published:** 2020-07-17

**Authors:** Guilherme A. Câmara, Milton Y. Nishiyama-Jr, Eduardo S. Kitano, Ursula C. Oliveira, Pedro I. da Silva, Inácio L. Junqueira-de-Azevedo, Alexandre K. Tashima

**Affiliations:** ^1^ Departamento de Bioquímica, Escola Paulista de Medicina, Universidade Federal de São Paulo, São Paulo, Brazil; ^2^ Laboratório Especial de Toxinologia Aplicada, Center of Toxins, Immune-Response and Cell Signaling, Instituto Butantan, São Paulo, Brazil; ^3^ Laboratory of Immunology, Heart Institute (InCor), Faculty of Medicine, University of Sao Paulo, Sao Paulo, Brazil

**Keywords:** *Acanthoscurria rondoniae*, cysteine-rich peptides, peptidomics, proteomics, multiomics, antimicrobial peptides, antiviral peptides, antitumoral peptides

## Abstract

The *Araneae* order is considered one of the most successful groups among venomous animals in the world. An important factor for this success is the production of venoms, a refined biological fluid rich in proteins, short peptides and cysteine-rich peptides (CRPs). These toxins may present pharmacologically relevant biological actions, as antimicrobial, antiviral and anticancer activities, for instance. Therefore, there is an increasing interest in the exploration of venom toxins for therapeutic reasons, such as drug development. However, the process of peptide sequencing and mainly the evaluation of potential biological activities of these peptides are laborious, considering the low yield of venom extraction and the high variability of toxins present in spider venoms. Here we show a robust methodology for identification, sequencing, and initial screening of potential bioactive peptides found in the venom of *Acanthoscurria rondoniae*. This methodology consists in a multiomics approach involving proteomics, peptidomics and transcriptomics analyses allied to *in silico* predictions of antibacterial, antifungal, antiviral, and anticancer activities. Through the application of this strategy, a total of 92,889 venom gland transcripts were assembled and 84 novel toxins were identified at the protein level, including seven short peptides and 10 fully sequenced CRPs (belonging to seven toxin families). *In silico* analysis suggests that seven CRPs families may have potential antimicrobial or antiviral activities, while two CRPs and four short peptides are potentially anticancer. Taken together, our results demonstrate an effective multiomics strategy for the discovery of new toxins and *in silico* screening of potential bioactivities. This strategy may be useful in toxin discovery, as well as in the screening of possible activities for the vast diversity of molecules produced by venomous animals.

## Introduction

Spider venoms are composed of a complex mixture of salts, nucleotides and other small molecules, as well as bioactive molecules such as proteins and peptides, usually referred to as toxins (Escoubas and Rash, 2004; Kuhn-Nentwig et al., 2011; Langenegger et al., 2019). In spiders, toxins are produced and stored in venom glands. These toxins are synthetized in an inactive form and undergo several maturation processes (i.e. signal-peptide cleavage, posttranslational modifications (PTMs) and disulfide-bond formation) before being secreted in its mature form (Mebs, 2001; Kaas and Craik, 2015).

The family of cysteine-rich peptides (CRPs) is the main class of toxins present in spider venoms, typically presenting molecular masses between 3 and 9 kDa. The toxins contain ≥6 cysteine residues that form disulfide-bonds (S-S), which confers high stability to the peptides (Fry et al., 2009; Undheim et al., 2015). CRPs acts in different voltage-gated ion channels (Kuhn-Nentwig et al., 2011; Langenegger et al., 2019), such as calcium (Kubista et al., 2007; Deng et al., 2014), potassium (Lee and MacKinnon, 2004; Liao et al., 2006; Lau et al., 2016) and sodium channels (Corzo et al., 2008; Rates et al., 2013; Zhou et al., 2020), making them valuable tools to investigate physiological processes (Ruta et al., 2003; Osteen et al., 2016). Moreover, through the modulation of these channels, spiders can induce paralysis in insects while having minor effects on other taxa, being potential lead molecules for the development of biopesticides (Windley et al., 2012; King and Hardy, 2013). Another class of spider toxins are the antimicrobial peptides (AMPs), commonly found in spider hemolymphs (Silva et al., 2000; Riciluca et al., 2012) as a component of innate immunity, but also found in spider venoms (Jung et al., 2006; Abreu et al., 2017). Usually, AMPs are small molecules rich in cationic and hydrophobic residues that fold into a cationic amphipathic secondary structure (Edwards et al., 2016). AMPs interacts with the negatively charged outer membrane of microorganisms (Seo et al., 2012) through nonspecific interactions with anionic lipids (Arouri et al., 2009), causing membrane disruption through different pore-forming mechanisms (Fuertes et al., 2011; Paredes-Gamero et al., 2012). Interestingly, anticancer peptides (ACPs) share the same main characteristics of AMPs, such as folding into a cationic amphipathic structure and interacting with the negatively charged outer-membrane (Gaspar et al., 2013). Therefore, there is an increasing interest in studying the application of AMPs for cancer treatment (Felício et al., 2017; Zhang et al., 2019; Pérez-Peinado et al., 2020).

The biochemical arsenal of spider venoms is essential not only in predation and self-defense, but also in feeding, mating and antimicrobial protection, among other possible roles (Schendel et al., 2019). Tarantula spiders are usually harmless to humans (Lucas et al., 1994; Vetter and Isbister, 2008), but their venoms are valuable natural sources of molecules with potential for biotechnological applications and pharmacological research (Escoubas and King, 2009; Mobli et al., 2017). According to the World Spider Catalog, there are more than 48,000 spider species described (http://wsc.nmbe.ch, accessed on May 1^st^, 2020) and it is estimated that they can produce more than 10 million bioactive toxins (Saez et al., 2010). However, according to the ArachnoServer 3.0 database, about 1,500 spider toxins are cataloged and curated to date (Pineda et al., 2018), representing a small fraction of the estimated universe of spider venom toxins. Thus, the discovery of biologically active peptides derived from spider venoms is still a promising field in toxinology research.

The advances in sensitivity and resolution of mass spectrometers as well as advances in DNA and RNA sequencing techniques have led to a remarkable increase in the number of toxins reported in the last years (Duan et al., 2013; Sanggaard et al., 2014; Zelanis and Keiji Tashima, 2014; Abreu et al., 2017; Zobel-Thropp et al., 2018; Langenegger et al., 2019). However, the increase in toxin identification is not synchronized with the functional description of new toxins, since the functional characterization involves a significant number of experimental processes. Advances in bioinformatics and computational capacity allowed the development of machine-learning algorithms that serve as useful allies in drug discovery (Kaas and Craik, 2015). These machine-learning based tools may be used to predict potential biological activities, such as antimicrobial (Meher et al., 2017) and antitumoral (Manavalan et al., 2017), based on the primary structure of toxins. Thus, they may serve as valuable guides in toxin selection for further investigation.

Our group developed a workflow based on transcriptomic analysis, multiple enzyme digestion of venoms, mass spectrometry and bioinformatic analysis focused on the full sequencing of mature toxins (Abreu et al., 2017; Lomazi et al., 2018). In a previous work, we completely sequenced and determined the number of S-S bonds of new mature CRPs from the venom of *Acanthoscurria gomesiana* (Abreu et al., 2017). In this present study, we used our methodology allied to *in silico* predictions of AMPs and ACPs to investigate the *A. rondoniae* venom, which to the best of our knowledge, remained largely unexplored to date.

## Materials and Methods

**Figure 1:**
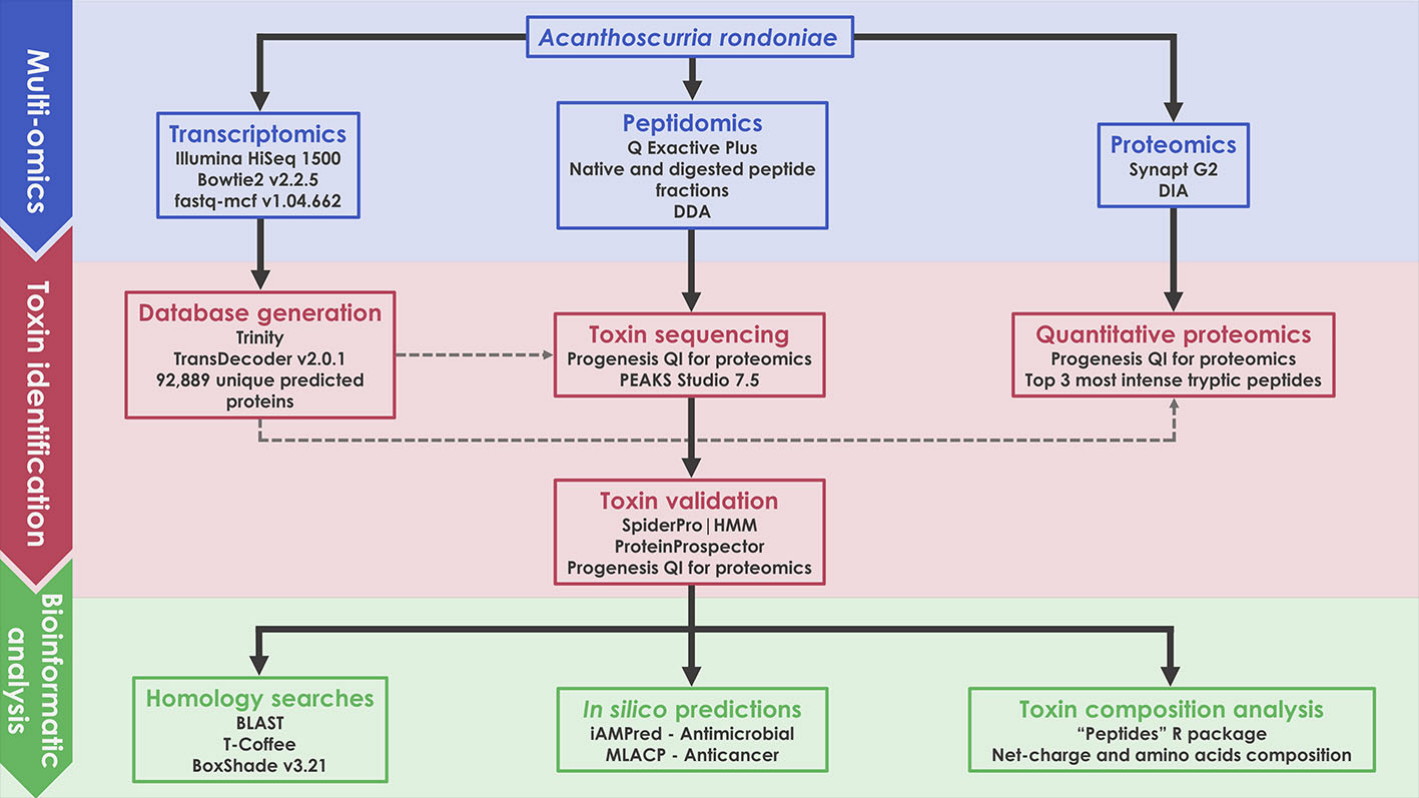
Schematic representation of the multiomics workflow applied in this work.

A scheme of the complete methodology used in this work is illustrated as a flow chart (**Figure 1**). Details of each block are given in the following items of this section.

### Reagents

All proteolytic enzymes were obtained from Promega (Madison, WI, USA). Dithiothreitol (DTT) and iodoacetamide (IAA) were acquired from GE Healthcare (Chicago, IL, USA). Acetonitrile (ACN) was purchased from Avantor (Center Valley, PA, USA). Unless otherwise stated, all other reagents were acquired from Sigma-Aldrich (Saint-Louis, MO, USA).

### Animals

Three adult *Acanthoscurria rondoniae* specimens were kept in captivity in the biotherium of the Laboratório Especial de Toxinologia Aplicada (LETA), Instituto Butantan (SP, Brazil). These animals were collected and maintained under SISBIO/ICMBio permanent license number 11024-3-IBAMA (Brazilian Institute of Environment and Renewable Natural Resources) and under the SisGen license number A82F014. The spiders were fed on 15-day intervals with cockroaches or crickets and had water *ad libitum*. All procedures were approved by the Research Ethical Committee of the Federal University of Sao Paulo (protocol number 7649061014).

### Venom Gland Transcriptome sequencing

One female specimen of *A. rondoniae* was anesthetized with carbon dioxide for about 10 min, euthanized to extract the venom glands, which were immediately stored at −80°C. mRNA from the venom glands was extracted and further processed for cDNA library construction following the stranded TruSeq RNA Sample Prep Kit protocol (Illumina, San Diego, CA, USA) (Freitas-de-sousa et al., 2015). Briefly, selected poly-A-RNA was fragmented and primed with random hexamers. Fragmented RNA was reverse transcribed and the generated first strand cDNA was ligated to indexing adapters for hybridization in the flow cell of a HiSeq 1500 System (Illumina, Inc) for sequencing. The size distribution of the cDNA libraries was measured by 2100 Bioanalyzer with DNA1000 assay (Agilent Technologies, CA, USA). An ABI Step One Plus Real-Time PCR System was used in quantification of the sample library before sequencing. The cDNA libraries were sequenced on the Illumina HiSeq 1500 System, in Rapid paired-end flow cell in a strategy of 300 cycles of 2*150bp paired-end. The RNA-seq raw sequencing reads were pre-processed through an “*in house*” pipeline for the detection of PhiX contaminant, using the software bowtie2 version 2.2.5 (Langmead and Salzberg, 2012), followed by filter quality control, to trim and remove reads with low-complexity and homopolymer enriched regions, poly-A/T/N tails, adapter sequences and low-quality bases with the software fastq-mcf 1.04.662 (Aronesty, 2013). Trimming was performed for reads shorter than 40bp and if mean quality score was lower than 25 in a window size equal to 15 and filtered out those composed by 90% of homopolymers or low-complexity regions. The raw data generated in this project was deposited in the NCBI BioProject section under the accession code PRJNA633430 and BioSample SAMN14943686. This Transcriptome Shotgun Assembly was deposited at NCBI TSA under the accession GIOJ00000000.

### Transcriptome Assembly and Database Generation

To generate a nonredundant set of transcripts, we performed a *de novo* assembly by the Trinity software (Grabherr et al., 2011) version r20131110, using 44,559,666 RNA-seq good quality paired-reads, with parameter CuffFly to reduce the number of false-positive isoforms, and minimum transcript length set to 300 bp. The prediction of translated amino acid sequences for the reconstructed transcripts were based on the TransDecoder software, version 2.0.1 (http://transdecoder.sourceforge.net/), considering only predicted proteins with protein length ≥60aa. Each transcript containing the coding sequences was aligned by BLASTp (Altschul, 1997) against the Uniprot/Swissprot protein database and the Transcriptome Shotgun Assembly (TSA) NCBI to assess the protein annotation with cutoff e-value <1e-5. The analysis of PFAM domains for the predicted proteins was based on hmmsearch tool in the software package hmmer (Johnson et al., 2010) against a PFAM domains database (Bateman, 2004), using the cutoff e-value <1e-3. The TransDecoder usually predicts more than one coding sequence by transcript and only one candidate was selected, following the priority order of match for UniProtKB/Swissprot, PFAM and TSA-NCBI for annotating and selecting the best candidate for each transcript.

### Venom Extraction and Enrichment

The venom extraction procedure was carried out as previously described (Rocha-e-Silva et al., 2009). Briefly, after one week of fasting, three *A. rondoniae* specimens were anesthetized with carbon dioxide (CO_2_) and had their venom glands electrically stimulated at a frequency of 10 Hz and voltage of 10–25 V. After extraction, the venom was pooled due to the low yield of each individual and then the pool was quantified using the Bradford reagent and bovine serum albumin (BSA) as a standard. After quantification, 500 µg of the pooled venom was submitted to solid-phase extraction using C18 StageTips (Rappsilber et al., 2007), with minor adjustments, in order to enrich the peptide fraction. Briefly, the StageTips were conditioned with 500 µl of methanol, then with 500 µl of methanol:water (1:1) and centrifuged at 2,100 rpm for 2 min in both steps. Then, 500 µl of 80% ACN in 0.1% trifluoroacetic acid (TFA) were added to the StageTips and centrifuged at 1,800 rpm for 3 min. In the following step, 500 µl of 5% ACN in 0.1% TFA were added and the tip was centrifuged for 1800 rpm for 3 min. After conditioning and stabilization, 500 µg of pooled venom (diluted in 5% ACN in 0.1% TFA, to a total volume of 500 µl) were loaded to the StageTip and then centrifuged for 3 min at 1,400 rpm. Three washing steps were performed using 500 µl of 5% ACN in 0.1% TFA and centrifugation at 1,700 rpm for 2 min. Lastly, the peptide fraction was eluted in 500 µl of 40% ACN in 0.1% TFA by centrifugation at 1,700 rpm for 3 min. After enrichment, the sample was divided into six aliquots of equal volumes (83 µl), vacuum-dried using a Concentrator Plus (Eppendorf) and stored at 4°C until the digestion step.

### Proteolytic Digestion

For proteomics analyses, a crude venom pool aliquot of 50 μg of proteins was digested with trypsin. For peptidomics analyses, five out of the six aliquots were submitted to proteolytic digestion using a different enzyme for each aliquot. The vacuum-dried aliquots were dissolved to 50 µl of digestion buffer according to the enzyme: NH_4_HCO_3_ 50 mM for trypsin and Asp-N; phosphate buffer 50 mM for Glu-C; Tris/HCl 100 mM, CaCl_2_ 10 mM for chymotrypsin and Tris/HCl 100 mM, CaCl_2_ 0.5 mM for thermolysin. The sixth aliquot was directly dissolved in 0.1% formic acid for LC-MS/MS analysis to characterize the toxins in their native (mature) forms.

To digest the toxins, volumes of 25 µl of 0.2% Rapigest surfactant (Waters, MA, USA) were added to each sample, which were incubated at 80°C for 15 min. Samples were reduced with 2.5 µl of DTT 100 mM for 30 min at 60°C and then alkylated with 2.5 µl of IAA 300 mM for 30 min at room temperature in the dark. After reduction and alkylation, the enzymes were added in an enzyme:protein ratio of 1:100 and incubated at 37°C for 30 min. Except for thermolysin, in which a ratio of 1:250 was used and the incubation was performed at 75°C for 15 min. TFA was added to a final concentration of 0.5% to stop the digestions. Samples were filtered using Ultrafree -MC PVDF 0,22 µm filters (Millipore), vacuum-dried and stored at −20°C until MS analysis.

### Mass Spectrometry: Peptidomics

For peptidomics analysis, digested and native peptide fractions were dissolved in 0.1% formic acid (solution A). Aliquots of 1 μl were automatically injected by a nano chromatography EASY-nlC 1200 system (Thermo Scientific) into a 15 cm x 50 µm Acclaim PepMap™ C18 column (Thermo Scientific) coupled to a Q Exactive Plus mass spectrometer (Thermo Scientific). Peptides were eluted with a linear gradient of 7%–45% of solution B (80% acetonitrile in 0.1% formic acid) at 300 nl/min for 60 min. Spray voltage was set at 2.5 kV and the mass spectrometer was operated in the data dependent mode, in which one full MS scan was acquired in the m/z range of 300-1,500 followed by MS/MS acquisition using higher energy collision dissociation (HCD) of the five most intense ions from the MS scan. MS and MS/MS spectra were acquired in the Orbitrap analyzer at 70,000 and 17,500 resolution (at 200 m/z), respectively. Unassigned and +1 charge states were not subjected to fragmentation. The maximum injection times and AGC targets were set to 25 ms and 3E6 for full MS, and 40 ms and 1E5 for MS/MS. The minimum signal threshold to trigger fragmentation event, isolation window and stepped normalized collision energy (NCE) were set to, respectively, 2.5E4 cps, 1.4 m/z and 26, 28, and 30. A dynamic peak exclusion was applied to avoid the same m/z selection for the next 5 seconds. All samples were analyzed in duplicates.

### Mass Spectrometry: Proteomics

The proteomics analysis of the digested crude venom pool was performed on a Synapt G2 mass spectrometer coupled to a nanoAcquity UPLC system (Waters). Five μl of peptide samples were loaded online in a Symmetry C18 trapping column (5 µm particles, 180 µm x 20 mm length; Waters) for 5 min at a flow rate of 8 µl/min of phase A (0.1% formic acid). The mixtures of trapped peptides were subsequently separated by elution with a gradient of 7%–35% of phase B (0.1% formic acid in acetonitrile) through a BEH 130 C18 column (1.7 µm particles, 75 mm by 150 mm; Waters) over 90 min at 275 nl/min. Data were acquired in the data independent acquisition mode HDMS^E^ with ion mobility separation in the m/z range of 50–2,000 and in the resolution mode. Peptide ions were fragmented by collision induced dissociation (CID) and energies were alternated between 4 eV and a ramp of 15–65 eV for precursor ion and fragment ions, respectively, using scan times of 1.25 s (Abreu et al., 2017; Pedroso et al., 2017). The ESI source was operated in positive mode with a capillary voltage of 3.0 kV, block temperature of 100°C, and cone voltage of 40 V. For lock mass correction, Glu-Fibrinopeptide B (500 fmol/mL in 50% acetonitrile, 0.1% formic acid; Peptide 2.0) was infused through the reference sprayer at 500 nl/min and sampled for 0.5 s every 60 s. The venom pool was analyzed in triplicate. All mass spectrometry data (DIA and DDA) were deposited to the ProteomeXchange Consortium *via* the PRIDE (Perez-Riverol et al. 2019) partner repository with the dataset identifier PXD019343.

### Bioinformatic Analysis

#### Quantitative Peptidomics and Proteomics

For quantitative peptidomics, mass spectrometry raw data of the native venom peptides were loaded in Progenesis QI for proteomics (Nonlinear Dynamics, Newcastle, UK). Briefly, a reference run for the duplicates was automatically selected. The retention times of precursor ions were processed for alignment, peak picking and normalized to the reference run using default parameters. The normalized data was exported from Progenesis QI for proteomics in.csv format and further analysis were made in Microsoft Excel (Microsoft), where precursor ions with an intensity below 5.0x10^5^ or with redundant m/z values were excluded from subsequent analysis.

Quantitative proteomics was also performed in Progenesis QI for proteomics with the same processing parameters. After processing, a.mgf file of all MS/MS spectra was exported to PEAKS Studio 7.5 (Bioinformatics Solutions Inc.) for protein identification (as described in “Toxin Sequencing”). The identification results were exported back to Progenesis as a.xml file. Venom proteins were quantified by the average signal intensity of the three most intense tryptic peptides of each protein (Silva et al., 2006). Only proteins identified with a minimum of three peptides and in at least two of the three replicates were considered for further analysis.

#### Toxin Sequencing

Mass spectrometry raw data of digested venom fractions were loaded and processed in PEAKS Studio 7.5 (Bioinformatic Solutions Inc.). *De novo* analysis was performed according to the following parameters: precursor ion mass tolerance of 10 ppm, fragment ion mass tolerance of 0.025 Da, maximum of one nonspecific cleavage, maximum of two missed cleavages and enzyme set according to the sample. Cys carbamidomethylation was set as fixed modification and Asn/Gln deamidation, Met oxidation and N-terminal acetylation as variable modifications. Database searches were performed with the same parameters of *de novo* analysis against the previously built *A. rondoniae* venom gland transcriptome (92,939 sequences and 251 common contaminants) utilizing *de novo* sequenced peptides with average local confidence (ALC) scores ≥50%. Posttranslational modifications and homology searches were performed through PEAKS PTM and SPIDER modules, respectively. The false discovery rate (FDR) was estimated by the decoy fusion method (Zhang et al., 2012) and set to a maximum of 1%.

#### Mature Toxin Validation

Primary structures of toxins identified by *de novo* and database search were submitted to analysis on the Spider|ProHMM module of ArachnoServer 3.0 (Pineda et al., 2018) in order to predict the cleavage sites of signal peptide and propeptide, ultimately resulting in the prediction of its mature sequences. The predicted mature sequences were then confronted with the sequences obtained experimentally, and if a correspondence was observed, the toxin was considered fully sequenced by LC-MS/MS. Mature sequences were also submitted to analysis in the MS-Product module of ProteinProspector v5.22 (http://prospector.ucsf.edu/prospector/mshome.htm), which provides theoretical fragmentations and precursor ion m/z values. The theoretical m/z values of precursor ions with charges ranging from +2 to +9 were compared to those assigned in Progenesis QI for proteomics and also manually validated in the raw data of the native toxins through Xcalibur (Thermo Scientific). If a peak corresponding to a m/z value of a mature toxin was found in the raw data and the consensus sequence was supported by MS/MS data, the presence of the toxin in the venom was validated.

### Homology Search and Alignment

Validated mature sequences were submitted to homology search through BLAST (https://www.uniprot.org/blast/), aligned with T-Coffee (http://tcoffee.crg.cat/apps/tcoffee/do:regular) and lastly, edited with Boxshade (http://www.ch.embnet.org/software/BOX_form.html).

### *In Silico* Anticancer and Antimicrobial Assays

For the prediction of possible biological activities, *in silico* analysis were performed by two tools. For antimicrobial activity, we utilized iAMPpred (Meher et al., 2017), a sequence-based computational tool which provides a score (ranging from 0 to 1) which reflects potential antibacterial, antiviral and antifungal properties. For this analysis, only scores >0.8 were considered as significant values. Gomesin from *A. gomesiana* hemolymph (Silva et al., 2000) was utilized as a positive control for antibacterial activity. Mouse β-defensin-4 (mBD4) and P9 (Zhao et al., 2016), a peptide derived from mBD4, were utilized as positive controls for antiviral activity. Rondonin from *A. rondoniae* hemolymph (Riciluca et al., 2012) and gomesin were utilized as positive controls for antifungal activity. For the prediction of antitumoral activities, we utilized MLACP (Manavalan et al., 2017), a sequence-based computational tool that utilizes two distinct machine-learning based algorithms, RFACP and SVMACP. Only peptides which presented a consensus with the two algorithms, with both presenting scores >0.5, were considered as potential ACPs, as recommended by the authors (Manavalan et al., 2017). For this analysis, we utilized gomesin (Ikonomopoulou et al., 2018), Aurein 1.2 from the frog *Litoria aurea* (Rozek et al., 2000) and human neutrophil peptide-1 (HNP-1) (Gaspar et al., 2015) as positive controls for anticancer activity.

### Net Charge and Amino Acids Composition Analysis

Net charge and amino acid composition were calculated by the R scripts in the package Peptides (Osorio et al., 2015). For net charge analysis, only CRPs were selected and Cys residues were not considered due to disulfide bonds. For amino acids composition, only predicted ACPs were selected.

## Results

### *A. rondoniae* Venom Gland Transcriptomics

Sequencing of the venom gland transcriptome of *A. rondoniae* resulted in a total 46,511,000 raw paired-reads. After the quality processing of raw reads, a total of 44,559,666 high-quality reads remained (95.8% of the total). *De novo* assembly using Trinity was performed on high-quality reads generating 150,409 transcripts, with an N50 value of 892 bp, a median length of 473 bp and an average transcript length of 735 bp. These transcripts generated 92,889 unique predicted proteins from the transcriptome, which were used as the reference database for the peptidomics and proteomics analysis.

### *A. rondoniae* Venom Peptidomics

**Figure 2:**
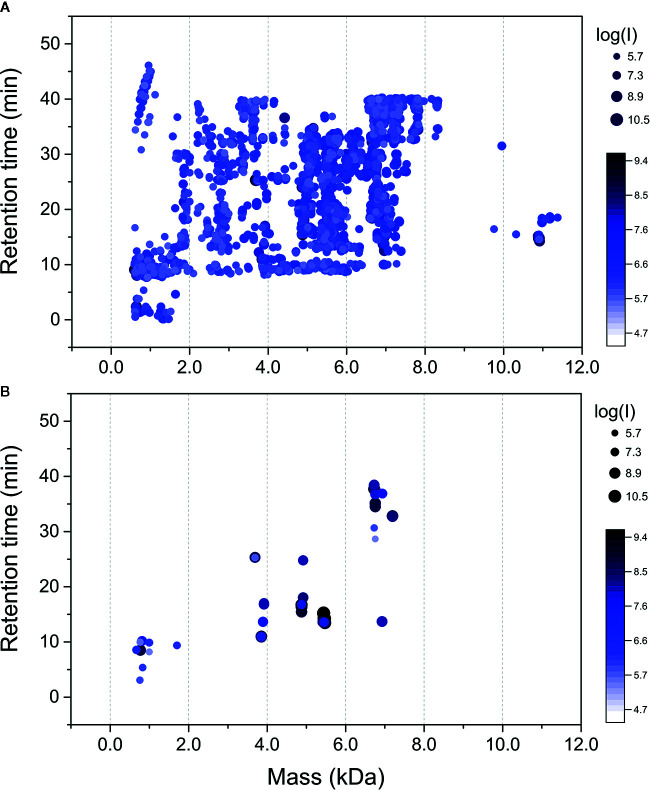
Mass maps of precursor ions identified in *Acanthoscurria rondoniae* peptidomic venom fraction after Progenesis QI for proteomics raw data processing. **(A)** 2,800 precursor ions obtained after manual filtering and clustering of redundant ions. **(B)** Precursor ions corresponding to the 18 mature toxins validated in *A. rondoniae* venom in different charge states.

Raw mass spectral files (.raw) of native venom peptides, were initially processed in Progenesis QI for proteomics, resulting in a total of 17,329 mature precursor ions recorded. After manual filtering for threshold establishment and clustering of redundant peptide ions, a total of 2,800 precursor ions were kept for further analysis (**Figure 2A** and **Supplementary Table 1**). Main clusters of precursor ions around 5, 5.5, 6, and 7 kDa, mass values typically observed for CRPs from tarantula venoms (King and Hardy, 2013; Abreu et al., 2017). Also, there is a cluster in a mass range below 1.5 kDa, which represents short peptides also commonly found in tarantulas (King and Hardy, 2013). *De novo* sequencing, database searches and homology analysis of MS/MS spectra of digested venom peptides resulted in the identification of 12,032 peptide spectrum matches corresponding to 2,770 cleaved peptides (**Supplementary Table 2**) belonging to 74 different proteins (**Supplementary Table 3**). Among these 74 proteins, 62 were identified with more than two unique peptides and 12 with two unique peptides. The N-terminal of the mature toxins was determined by our multiple digestion approach, given that the same N-terminal amino acid was identified by consensus MS/MS spectra from different enzymes. Although the solid phase extraction step of our method was focused to enrich venom peptides, we identified 55 proteins with mass above 15 kDa (**Supplementary Table 3**). However, the protein masses from entries in the transcriptomic database are from the complete sequences, with the signal peptide and prodomains, adding the respective masses to the mature proteins. Besides, part of the heavier venom proteins may have bypassed the enrichment, as it seems to be the case of the 53.3 kDa Ar-CRISP, identified with 202 unique peptides and 80% coverage, and the 82.4 kDa Ar-Neprilysin-1, identified with 193 unique peptides and 81% coverage (**Supplementary Table 3**).

The theoretical m/z values of these 74 proteins were calculated in ProteinProspector and then compared to those present in Progenesis QI for proteomics data (**Supplementary Table 1**). A total of 57 toxins fully sequenced from the overlapping peptide fragments generated by multiple enzyme digestions are possible mutated CRPs and posttranslational modified forms. To validate the identification of mature toxins, precursor ion spectra were manually analyzed in Xcalibur (Thermo Scientific) to confirm monoisotopic peak and charge state assignments. This resulted in the validation of 17 new toxins and also of the U1-TRTX-Agm3a, a toxin described by our group in the *Acanthoscurria gomesiana* venom (Abreu et al., 2017). In total, 18 mature toxins were validated (**Figure 2B** and **Table 1**). The 17 new toxins are distributed in 7 families containing a total of 10 CRPs and seven short peptides (**Table 1**). The CRPs families were named according to the nomenclature proposed by King et al. (King et al., 2008). In order to add another level of validation to the mature CRPs identified, the whole translated sequences predicted by our transcriptome were processed in the SpiderPro|HMM module of ArachnoServer 3.0 (Pineda et al., 2018). The mature sequences of six CRP families were confirmed by the propeptide and signal peptide cleavage site predictions, here represented by the predicted transcript of each family, except for the U6-TRTX-Ar1a, which is two amino acids residues (NR) longer than predicted in the N-terminus (**Supplementary Table 4**).

**Table 1:** Mature toxins identified and validated in *Acanthoscurria rondoniae* peptidomic venom fraction.

Toxin	Monoisotopic mass (Da)	Mature sequence	AA	S-S	Log(I)	Rank
VLPVFV	670.41	PLPVFV	6	–	6.38	15
VVVPFVV	757.47	VVVPFVV	7	–	8.54	9
VENLAEP	770.39	VENLAEP	7	–	8.78	8
VLPPLKF	812.40	VLPPLKF	7	–	7.60	13
VPPILKY	828.51	VPPILKY	7	–	5.91	18
YPPPPPPPH	997.50	YPPPPPPPH	9	–	6.21	16
FETPNNPDGKVTKQE	1702.82	FETPNNPDGKVTKQE	15	–	6.08	17
U1-TRTX-Agm3a	3690.49	ACGSFMWKCSERLPCCQEYVCSPQWKWCQNP	31	3	9.49	2
U1-TRTX-Ar1a	3852.54	SCVHERETCSKVRGPLCCRGECTCPIYGDCFCYGS	35	4	9.08	4
U1-TRTX-Ar1b	3920.58	SCVYERETCSKVRGPLCCRGECTCPIYGDCFCYGS	35	4	8.05	12
U2-TRTX-Ar1a	4876.02	CATENVPCDENRPGDCCSEYECLKPTGHGWWYASYYCYKKKSG	43	3	8.91	6
U3-TRTX-Ar1a	5439.80	IIECFFSCEIEKDGKSKEGKPCKPKGDKDKDKKCSGGWRCKLKLCLKI	48	3	10.29	10
U3-TRTX-Ar1b	5457.75	IIECFFSCEIEKDGKSKEGKPCKPKGDKDKDKKCSGGWRCKLKMCLKI	48	3	10.54	1
U4-TRTX-Ar1a	6728.19	ECKQLKEKCSNHCDCCGKTVLCATVYVGRNTEMLCKEKRSDDPILNSIGKVINAATKAMSGC	62	4	9.36	3
U4-TRTX-Ar1b	6755.19	ECKQLKEKCNNHCDCCGKTVLCATVYVGRNTEMLCKEKRSDDPILNSIGKVINAATKAMSGC	62	4	8.80	7
U5-TRTX-Ar1a	6928.76	ACTTEADCPNGCCTGGSFHRYCRSYGGEMDQCEPRNDFGSYSTACPCKEEFECSPIKRCQRR	62	5	8.32	11
U6-TRTX-Ar1a	6936.18	NREHCYIPRRRCVTTEQCCKPYDTVNYFVACGKAWPEDKKRKVNKCYICNNELTVCTR	58	4	7.56	14
U7-TRTX-Ar1a	7195.23	ETSCIEELQTCKNSCECCGTTTICSPSWVDGNEIKLCRNEGNKLQKVWHFFQKAYSKMHSCKT	63	4	8.98	5

Intensities are shown in a logarithmic scale (log10) and ranks were assigned based on the intensities. AA, number of amino acids; S-S, number of disulfide bonds.

We could not validate the presence of the mature forms of the other 40 possibly mutated or modified CRPs and thousands of native precursor ions still remained to be identified. We consider that many of these forms are derived from the main CRP families identified, as clusters of masses are observed around the main seven classes reported here (**Figure 2A** and **Table 1**). Posttranslational modifications, mutations and proteolytic processing at alternative sites may result in a complex population of toxin proteoforms present in the spider venoms. Incorrect assignment of monoisotopic peaks and charge state on acquisition may also limit identification and redundant ion clustering. In addition, native toxins of ~11 kDa (**Figure 2A**) could not have their mature forms identified and other CRP families may have been missed in our native peptidomic analysis. For *in silico* analysis, only the 18 validated toxins without any posttranslational modifications were utilized.

### *A. rondoniae* Venom Proteomics

In the proteomics analysis of the *A. rondoniae* venom 33 proteins were quantified. We only considered proteins identified with at least three peptides and present in two out of three replicates (**Table 2**). The most abundant venom protein was the cysteine-rich secretory protein (Ar-CRISP), composing 28% of the venom, followed by the CRP U3-TRTX-Ar1a (26%) and then, the U5-TRTX-Ar1a (15%). These first three toxins represent 69% of the *A. rondoniae* venom toxins. The CRP proteoforms identified in the peptidomics analysis were not included in the quantitative proteomics due to the difficulty to precisely quantify sequences with high homologies. But the U3-TRTX-Ar1b, differing by only one amino acid from the U3-TRTX-Ar1a (L44M), is the most intense peak among the native CRPs, corroborating the proteomics results. The most abundant toxin family is of the CRP, composing 58% of the venom (**Table 2**). The venom also contains significant amounts of the metalloprotease neprilysin-1 (Ar-Neprilysin-1, 8.4%) and hyaluronidase (Ar-Hyaluronidase, 1.5%). The proteins are homologous to those of the *A. geniculata* venom (Sanggaard et al., 2014). The Ar-CRISP is 76% homologous to the putative cysteine-rich protease (L1941_T1/1_Tarantula_S_fr3), Ar-Neprilysin-1 is 74% and Ar-Hyaluronidase is 80% to the respective Membrane venom metalloendopeptidase-a (L67_T1/2_Tarantula_V_fr5) and Venom hyaluronidase (L1941_T1/1_Tarantula_S_fr3) from *A. geniculata* (Sanggaard et al., 2014).

**Table 2:** Relative quantification of proteins by proteomic analysis of the venom of *Acanthoscurria rondoniae*.

Accession	Peptide count	Mass (kDa)	Description	NI	%
comp117964_c0_seq1.p1	181	53.3	Ar-CRISP	1.38E+07	27.9%
comp90511_c0_seq1.p1	28	10.7	U3-TRTX-Ar1a	1.29E+07	25.9%
comp105543_c0_seq1.p3	25	9.4	U5-TRTX-Ar1a	7.29E+06	14.7%
comp127239_c0_seq1.p1	101	82.4	Ar-Neprilysin-1	4.16E+06	8.4%
comp57753_c0_seq1.p1	13	12.3	U4-TRTX-Ar1a	4.13E+06	8.3%
comp90508_c2_seq2.p1	5	15	U2-TRTX-Ar1a	2.51E+06	5.1%
comp107050_c0_seq2.p2	12	13	U7-TRTX-Ar1a	1.47E+06	3.0%
comp127127_c4_seq1.p1	46	49.8	Ar-Hyaluronidase	7.49E+05	1.5%
comp125618_c0_seq2.p1	5	6.9	U1-TRTX-Agm3a	6.81E+05	1.4%
comp90482_c0_seq1.p1	26	48.6	PFAM: Serpin (serine protease inhibitor)	4.25E+05	0.9%
comp98439_c0_seq1.p1	4	16.3	PFAM: Thyroglobulin type-1 repeat	3.23E+05	0.7%
comp117041_c0_seq1.p1	28	42.8	PFAM: Tyrosine phosphatase family	2.45E+05	0.5%
comp57865_c0_seq1.p1	7	18.5	Unknown	1.86E+05	0.4%
comp99029_c0_seq1.p1	4	10.8	U6-TRTX-Ar1a	1.62E+05	0.3%
comp117273_c1_seq1.p1	23	45.1	PFAM: Putative serine esterase (DUF676)	1.46E+05	0.3%
comp119317_c0_seq2.p1	20	87.2	PFAM: Peptidase family M13	1.28E+05	0.3%
comp116850_c0_seq2.p1	9	53.1	PFAM: Zinc carboxypeptidase	6.93E+04	0.1%
comp122884_c0_seq2.p1	5	78.9	PFAM: Peptidase S8 pro-domain	4.99E+04	0.1%
comp117075_c0_seq1.p1	3	25.5	PFAM: Tetraspanin family	4.61E+04	0.1%
comp115582_c0_seq1.p1	10	76.4	PFAM: Neutral/alkaline nonlysosomal ceramidase	4.30E+04	0.1%
comp113427_c0_seq1.p1	11	91.1	PFAM: Fasciclin domain	4.15E+04	0.1%
comp87917_c0_seq1.p1	5	12.2	TSA: U3-hexatoxin-Hib [Hadronyche infensa]	1.98E+04	0.0%
comp27569_c0_seq1.p1	3	27.4	TSA: putative uncharacterized protein. partial	1.53E+04	0.0%
comp126642_c0_seq1.p1	3	46.1	PFAM: Cysteine-rich secretory protein family	1.16E+04	0.0%
comp114378_c0_seq1.p1	18	72.2	PFAM: Hemocyanin. copper containing domain	7.96E+03	0.0%
comp120272_c1_seq8.p1	3	39.9	TSA: peptidylglycine alpha-hydroxylating monooxygenase	4.76E+03	0.0%
comp121853_c0_seq2.p1	6	72.3	PFAM: Angiotensin-converting enzyme	4.54E+03	0.0%
comp114378_c0_seq4.p1	17	71.5	PFAM: Hemocyanin. copper containing domain	2.72E+03	0.0%
comp117984_c0_seq2.p1	4	31.1	PFAM: Immunoglobulin I-set domain	2.60E+03	0.0%
comp57921_c1_seq1.p1	4	44.9	PFAM: Hemocyanin. ig-like domain	7.99E+02	0.0%
comp114578_c0_seq3.p1	7	25.1	TSA: tri-cap-1 [Trittame loki]	4.91E+02	0.0%
comp114378_c0_seq2.p1	16	73.7	PFAM: Hemocyanin. ig-like domain	1.71E+02	0.0%
comp86283_c0_seq1.p1	6	78.4	PFAM: Transferrin	0.00E+00	0.0%

### *In Silico* Analysis Suggests Possible Antimicrobial Activities of CRPs and Antitumoral Activities of Short Peptides

For screening of the possible biological activities of *A. rondoniae* toxins, we performed *in silico* simulations using iAMPred (Meher et al., 2017) and MLACP (Manavalan et al., 2017), two machine-learning algorithms to evaluate potential antimicrobial and antitumoral activities, respectively. Both tools give as the output a score from 0 to 1. Higher scores suggest a higher probability of presenting the respective activity.

The results indicate that all new CRPs may have antimicrobial activities (**Table 3**). In general, higher scores were obtained for antimicrobial and antifungal activities (>0.9 for both in all seven families), but all CRPs showed antiviral scores >0.5, with the lowest score being 0.685 for U2-TRTX-Ar1a, while all other toxins had scores higher than 0.8. The U3-TRTX-Ar1x family demonstrated the highest scores for antibacterial (>0.99) and antifungal activities (>0.97). As for short peptides, our results suggest a lower probability of presenting antimicrobial activity, except for the peptide VLPPLKF, which had scores above 0.79 for all three activities. Our positive control for antibacterial activity, gomesin, obtained a score of 0.985, close to those observed for the seven CRPs families. This was also observed for the positive controls of antifungal activity, gomesin and rondonin, with scores of 0.973 and 0.903, respectively (**Table 3**). Lastly, our positive controls for antiviral activity, mBD4 and P9, a peptide derived from mBD4, obtained scores of 0.773 and 0.940, respectively.

**Table 3:** *In silico* predictions of biological activities of validated toxins identified in the venom of *A. rondoniae* spiders.

	Antimicrobial activity				Antitumoral activity			
Peptide	Bacterial	Viral	Fungal		RFACP	Prob.	SVMACP	Prob.
Gomesin	0.985	0.898	0.973		ACP	0.668	ACP	0.926
Rondonin	0.671	0.301	0.903		Non-ACP	0.400	Non-ACP	0.447
P9	0.997	0.940	0.992		Non-ACP	0.479	ACP	0.816
mBD4	0.990	0.773	0.968		Non-ACP	0.371	ACP	0.837
Aurein 1.2	0.940	0.913	0.917		ACP	0.870	ACP	0.935
HNP-1	0.920	0.920	0.950		ACP	0.879	ACP	0.938
PLPVFV	0.623	0.586	0.515		ACP	0.639	ACP	0.656
VVVPFVV	0.645	0.538	0.432		ACP	0.637	ACP	0.635
VENLAEP	0.083	0.046	0.028		Non-ACP	0.296	Non-ACP	0.508
VLPPLKF	0.801	0.794	0.828		ACP	0.632	ACP	0.740
VPPILKY	0.751	0.410	0.429		ACP	0.501	ACP	0.528
YPPPPPPPH	0.542	0.382	0.406		Non-ACP	0.442	ACP	0.618
FETPNNPDGKVTKQE	0.133	0.143	0.082		Non-ACP	0.350	Non-ACP	0.164
U1-TRTX-Agm3a	0.843	0.696	0.579		ACP	0.559	ACP	0.878
U1-TRTX-Ar1a	0.994	0.968	0.976		Non-ACP	0.489	ACP	0.862
U1-TRTX-Ar1b	0.987	0.966	0.972		ACP	0.541	ACP	0.903
U2-TRTX-Ar1a	0.940	0.685	0.920		Non-ACP	0.283	ACP	0.624
U3-TRTX-Ar1a	0.996	0.918	0.982		Non-ACP	0.292	Non-ACP	0.360
U3-TRTX-Ar1b	0.997	0.924	0.986		Non-ACP	0.324	Non-ACP	0.381
U4-TRTX-Ar1a	0.981	0.770	0.954		Non-ACP	0.139	Non-ACP	0.121
U4-TRTX-Ar1b	0.978	0.742	0.937		Non-ACP	0.142	Non-ACP	0.128
U5-TRTX-Ar1a	0.996	0.858	0.989		Non-ACP	0.363	ACP	0.519
U6-TRTX-Ar1a	0.986	0.826	0.947		Non-ACP	0.300	ACP	0.538
U7-TRTX-Ar1a	0.991	0.855	0.926		Non-ACP	0.256	Non-ACP	0.302

Antimicrobial activities were predicted by iAMPpred (Meher et al., 2017). Anticancer activities were predicted by MLACP (Manavalan et al., 2017): RFACP, random forest method; SVAMCP, support vector machine method; ACP, anticancer peptide; Non-ACP, nonanticancer peptide.

On the other hand, the results obtained for antitumoral activities demonstrate that short peptides of *A. rondoniae* are more prone to present antitumoral properties than the CRPs in this *in silico* analysis. From the 7 short peptides, 4 demonstrated a consensus on the two algorithms, indicating potential antitumoral activities (**Table 3**). These short peptides are: PLPVFV, VPPILKY, VVVPFVV and VLPPLKF. The two CRPs indicating potential antitumoral activity are the U1-TRTX-Agm3a and the U1-TRTX-Ar1b. Gomesin, aurein 1.2 and human neutrophil peptide-1 (HNP-1), used as positive controls for anticancer activity, demonstrated consensus on the two algorithms (**Table 3**).

### Homology Analysis Indicates Potential Biological Activities for Cysteine-Rich Toxins

**Figure 3:**
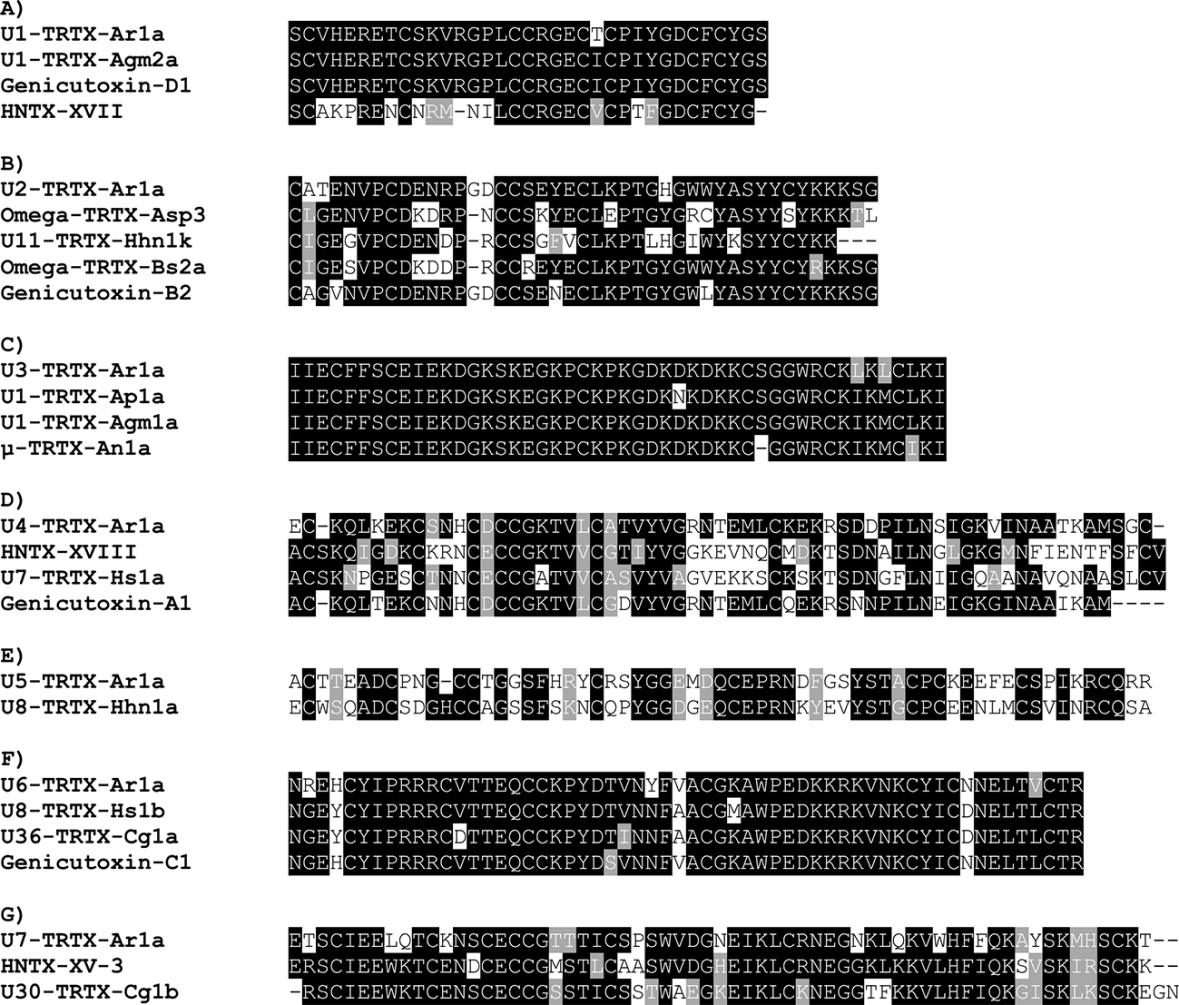
Alignments of the seven families of CRPs validated in *Acanthoscurria rondoniae* peptidomic venom fraction with homologous toxins (>50% primary structure similarity) from other spider species. **(A)** U1-TRTX-Ar1a: *A. rondoniae*; U1-TRTX-Agm2a: *A. gomesiana* (UniProt ID: P0DQJ4); Genicutoxin-D1: *A. geniculata*; HNTX-XVII: *Cyriopagopus hainanus* (UniProt ID: D2Y2C5). **(B)** U2-TRTX-Ar1a: *A. rondoniae*; Omega-TRTX-Asp3: *Aphonopelma sp* (UniProt ID: P0CI04); U11-TRTX-Hhn1k: *Cyriopagopus hainanus* (UniProt ID: D2Y281); Ω-TRTX-Bs2a: *Brachypelma smithi* (UniProt ID: B3FIV1); Genicutoxin-B2: *A. geniculata*. **(C)** U3-TRTX-Ar1a: *A. rondoniae*; U1-TRTX-Ap1a: *A. paulensis* (UniProt ID: B3EWY4); U1-TRTX-Agm1a: *A. gomesiana* (UniProt ID: P0DQJ3); μ-TRTX-An1a: *A. natalensis* (UniProt ID: B3A0P0). **(D)** U4-TRTX-Ar1a: *A. rondoniae*; HNTX-XVIII: *Cyriopagopus hainanus* (UniProt ID: D2Y251); U7-TRTX-Hs1a: *Cyriopagopus schmidti* (UniProt ID: B3FIN4); Genicutoxin-A1: *A. geniculata*. **(E)** U5-TRTX-Ar1a: *A. rondoniae*; U8-TRTX-Hhn1a: *Cyriopagopus hainanus* (UniProt ID: D2Y2C0). **(F)** U6-TRTX-Ar1a: *A. rondoniae*; U8-TRTX-Hs1b: *Cyriopagopus schmidti* (UniProt ID: P82960); U36-TRTX-Cg1a: *Chilobrachys guangxiensis* (UniProt ID: B1P1J5); Genicutoxin-C1: *A. geniculata*. **(G)** U7-TRTX-Ar1a: *A. rondoniae*; HNTX-XV-3: *Cyriopagopus hainanus* (UniProt ID: D2Y2B9); U30-TRTX-Cg1b: *Chilobrachys guangxiensis* (UniProt ID: B1P1I2).

In order to identify homologous toxins from other spiders and provide insights of possible biological activities by structural similarity, one toxin of each of the seven families of CRPs were selected for BLAST analysis. In general, our results demonstrated a high conservation of primary sequences among species of the same genus (Acanthoscurria) and decreasing homology with increasing distance in the phylogenetic tree (**Figure 3**). For instance, the U1-TRTX-Ar1a is 97% homologous to the *A. gomesiana* U1-TRTX-Agm2a (Abreu et al., 2017) and to the *A. geniculata* genicutoxin-D1 (Sanggaard et al., 2014). The next closest homolog is the HNTX-XVII.3, from *Cyriopagopus hainanus* (**Figure 3A**), with 62% homology. U1-TRTX-Agm2a has potential antimicrobial activity (Abreu et al., 2017) and HNTX-XVII.3 presents weak inhibition of Kv1.2/KCNA2 and Kv1.3/KCNA3 voltage-gated potassium channels. U1-TRTX-Ar1a presented high levels of *in silico* antibacterial, antiviral and antifungal scores (>0.96 for all three) and may present potassium channel modulation by homology to the HNTX-XVII.3.

The U2-TRTX-Ar1a is 88% homologous to the genicutoxin-B2 from *A. geniculata* (Sanggaard et al., 2014), 74% to Ω-TRTX-Bs2a from *Brachypelma smithi* (Corzo et al., 2008) (**Figure 3B**), 70% to U11-TRTX-Hhn1k from *Cyriopagopus hainanus* and 69% to Ω-TRTX-Asp3, from *Aphonopelma* sp. *Brachypelma smithi* is a Mexican Theraphosid and the Ω-TRTX-Bs2a has insecticidal activity against crickets, possibly acting on the Para/tipE insect sodium channels (Corzo et al., 2008). As the U2-TRTX-Ar1a, the Ω-TRTX-Bs2a is amidated at the C-terminal Ser. Interesting to notice that the identification of a peptidylglycine alpha-hydroxylating monooxygenase in the venom proteome (comp120272_c1_seq8.p1, **Table 2**) supports this PTM. The U3-TRTX-Ar1a is highly homologous to toxins already reported in other Acanthoscurria spiders, with 96% homology to the *A. gomesiana* U1-TRTX-Agm1a (Abreu et al., 2017), 94% to the *A. paulensis* U1-TRTX-Ap1a (Mourão et al., 2013) and 92% to the *A. natalensis* µ-TRTX-An1a (Rates et al., 2013) (**Figure 3C**). The U3-TRTX-Ar1a levels of antimicrobial scores were >0.98 for antibacterial and antifungal activities, corresponding to the antimicrobial potential of U1-TRTX-Agm1a (Abreu et al., 2017). The U1-TRTX-Ap1a is insecticidal against *Spodoptera frugiperda* caterpillars and *Drosophila melanogaster* (Mourão et al., 2013). And the µ-TRTX-An1a affects insect neuronal voltage-dependent sodium channels (Rates et al., 2013). The U4-TRTX-Ar1a, U5-TRTX-Ar1a, U6-TRTX-Ar1a and U7-TRTX-Ar1a presented different levels of homology to other spider toxins reported mostly at the transcript level (**Figure 3**).

### Net Charge Analysis and Amino Acid Composition

**Figure 4:**
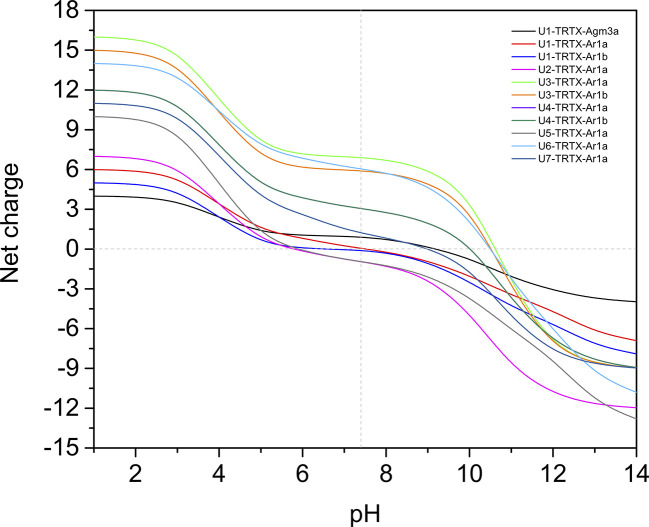
Net-charge analysis of all CRPs validated in *Acanthoscurria rondoniae* peptidomic venom fraction. Net charges were calculated in pH ranges of 0-14 in 0.5 intervals.

From all 11 CRPs analyzed, seven presented positive net charge at physiological pH (7.4), two are negatively charged and two are neutral (**Figure 4**). The U6-TRTX-Ar1a presented the highest net charge at physiological pH (+6,0), followed by the U3-TRTX-Ar1a/b with a net charge of +5,9. The negatively charged CRPs U2-TRTX-Ar1a and U5-TRTX-Ar1a presented net charges values of –0,96, and −0,95, respectively. As for amino acid composition, only the predicted ACPs were selected, totalizing two CRPs and four short peptides. Our main goal was to evaluate the percentage of nonpolar (hydrophobic) residues in those toxins. We observed high percentages of hydrophobic residues (>60%) in CRPs and even higher percentages of hydrophobic residues (>85%) in short peptides (**Table 4**).

**Table 4:** Amino acids composition analysis of anticancer peptides (ACPs) predicted by MLACP (Manavalan et al., 2017).

Amino acids class	Peptide											
	U1-TRTX-Agm3a		U1-TRTX-Ar1b		VLPPLKF		PLPVFV		VVVPFVV		VPPILKY	
	#	%	#	%	#	%	#	%	#	%	#	%
Tiny	5	20	9	33.3	0	0	0	0	0	0	0	0
Small	10	40	14	51.9	3	42.9	4	66.7	6	85.7	3	42.9
Aliphatic	3	12	4	14.8	3	42.9	3	50	5	71.4	3	42.9
Aromatic	5	20	4	14.8	1	14.3	1	16.7	1	14.3	1	14.3
Hydrophobic	13	52	14	51.9	6	85.7	6	100	7	100	6	85.7
Hydrophilic	12	48	13	48.1	1	14.3	0	0	0	0	1	14.3
Charged	5	20	8	29.6	1	14.3	0	0	0	0	1	14.3
Cationic	3	12	4	14.8	1	14.3	0	0	0	0	1	14.3
Anionic	2	8	4	14.8	0	0	0	0	0	0	0	0

## Discussion

In this study, we applied a multiomics strategy to explore the venom composition of *A. rondoniae* and *in silico* analysis in order to prospect new toxins with possible therapeutical applications. Previously, one experimental study was conducted to characterize AMPs from *A. rondoniae* spiders, which led to the identification of rondonin, an antifungal peptide present in the spider hemolymph (Riciluca et al., 2012). To our knowledge, this work is the first analysis of *A. rondoniae* venom composition. Through this strategy, we sequenced the venom gland transcriptome, identified and quantified proteins and determined the sequences of mature toxins of 17 new CRPs and short peptides present in the native venom, as well as one previously identified by our group, U1-TRTX-Agm3a (Abreu et al., 2017).

Homology searches and alignments demonstrated similarities of all seven CRP families to toxins reported in other spider venoms and, as expected, higher similarity to toxins of other Acanthoscurria spiders. The results indicate that these toxins may be biologically essential for the spider survival and also highlights a close phylogeny relationship within the genus. The U3-TRTX-Ar1x family demonstrated a high similarity with the toxins U1-TRTX-Ap1a, U1-TRTX-Agm1a, and µ-TRTX-An1a, all from other Acanthoscurria spiders. It is important to notice that this family also corresponds to the most expressed CRP in *A. rondoniae* venom observed by the quantitative proteomics and peptidomics approaches. The data highlights the relevance of U3-TRTX-Ar1x family for *A. rondoniae* spiders. Similarly to other spider venoms, enzymes as neprilysin, hyaluronidase, and carboxypeptidases, among others were found in the venom of *A. rondoniae* (Sanggaard et al., 2014; Borges et al., 2016; Kuhn-Nentwig et al., 2019). These enzymes may act in synergy with the neurotoxic CRPs to increase the spread and efficiency of the venom in the preys, as hypothesized elsewhere (Kuhn-Nentwig et al., 2019).

Our results from antimicrobial activity prediction demonstrated that all new CRPs identified in *A. rondoniae* venom have a probability of being antimicrobial, while only one short peptide (sequence: VLPPLKF) demonstrated possible antibacterial and antifungal properties. Gomesin was used as a positive control for antibacterial score since it has shown experimental activity against several Gram-positive and Gram-negative bacteria, such as *Escherichia coli*, *Klebsiella pneumoniae*, *Bacillus spp* and *Staphylococcus spp* (Silva et al., 2000). Gomesin scored 0.985 in iAMPpred for antibacterial activity, which is slightly lower than most of CRPs analyzed (**Table 3**). As for antifungal activities, gomesin and rondonin were selected as positive controls. Gomesin demonstrated activity against the filamentous fungi *Tricoderma viridae* as well as the yeast *Candida albicans* (Silva et al., 2000), while rondonin demonstrated activity against *Candida albicans* (Riciluca et al., 2012). The scores for antifungal activity obtained for gomesin and rondonin were 0.973 and 0.903, respectively, while all seven families of CRPs presented antifungal scores >0.92. Taken together, these results suggest the probabilities of the new CRPs identified in this work to present antibacterial and antifungal activities, which should be further explored by *in vitro* and *in vivo* assays. From all seven families of new CRPs, the U3-TRTX-Ar1x family presented the highest scores for antibacterial (>0.99) and antifungal (>0.98) activities, probably due to the high proportion of basic residues (**Table 1**), which impacts directly on net-charge and isoelectric points. These toxins have a net-charge of 5.94 at pH 7.4 (**Figure 4**). The high positive net charges are relevant and possibly increase antimicrobial activity, as evidenced by other studies with cationic peptides (Jiang et al., 2008; Paredes-Gamero et al., 2012). It is also important to notice that these toxins present high isoelectric points of 10.60 and 10.52 for U3-TRTX-Ar1and U6-TRTX-Ar1a, respectively (**Figure 4**). The net positive charges at neutral pH probably increase the efficiency of interaction with negatively charged membranes of microorganisms (Jiang et al., 2008). Future experiments may confirm the antimicrobial activity of the *A. rondoniae* peptides.

Many of the CRPs presented *in silico* antiviral scores, although in lower levels than antifungal and antibacterial, on average (**Table 3**). For antiviral activity prediction, we utilized mBD4 and P9, a peptide derived from mBD4, which has shown a broad activity of antiviral effects on respiratory virus such as H1N1, H3N2, H5N1, H7N7, H7N9, SARS-CoV and MERS-CoV in *in vivo* and *in vitro* assays (Zhao et al., 2016). The scores for antiviral activity of mBD4 and P9 were 0.773 and 0.940, respectively. The toxins from the U1-TRTX-Ar1x family demonstrated higher scores than P9 for antiviral activity, while all other families except U2-TRTX-Ar1x and U4-TRTX-Ar1x demonstrated scores between those obtained from mBD4 and P9, which also suggests a potential antiviral activity and should be further evaluated by *in vitro* and *in vivo* assays. Antiviral peptides may be promising therapeutic drugs (Vilas Boas et al., 2019). Some Arthropod peptides were found to suppress viral gene expression, as the cecropin A from the moth *Hyalophora cecropia*, which inhibited HIV activity (Wachinger et al., 1998), and mucroporin-M1, a peptide derived from the venom of the scorpion *Lychas mucronatus* which inhibited the activities of measles, SARS-CoV and influenza H5N1 viruses (Li et al., 2011). The authors proposed that the antiviral action of the peptide mucroporin-M1 could be by interaction with the virus envelope, binding to it by surface charge interactions and drastically decreasing the infectivity of the three viruses (Li et al., 2011). Several of the *A. rondoniae* CRPs found in this work present positive net charges at physiological pH and could be promising antiviral peptides, although this is not the only property to be considered.

In regard to *in silico* antitumoral activities, the predictions suggest that only two CRPs, U1-TRTX-Ar1b and U1-TRTX-Agm3a, may present antitumoral properties, as well as four short peptides: VLPPLKF, PLPVFV, VVVPFVV and VPPILKY. For this analysis, we utilized HNP-1 and Aurein 1.2 as positive controls. HNP-1, an human α-defensin AMP, showed cytotoxic activity against prostate tumor cells *in vitro* (Gaspar et al., 2015). Aurein 1.2, derived from the frog *Litoria aurea*, is another example of AMP with anticancer activity, as demonstrated by *in vitro* assays (Rozek et al., 2000). Both controls scored >0.84 in both algorithms of MLACP and, consequently, were predicted as ACPs. From all 11 CRPs, U1-TRTX-Agm3a and U1-TRTX-Ar1b have the shortest amino acids sequences, with 31 and 35 amino acids, respectively. It is also important to highlight that hydrophobicity plays a pivotal role in ACPs activity (Huang et al., 2011) and, as shown in **Table 4**, these potential ACPs are composed of more than 50% hydrophobic residues. It is interesting to note that short peptides indicated potential anticancer activity. In a possible peptide therapy, the potential of short peptides may be advantageous as they are easier to synthesize and modify, present higher ability to penetrate tumors and good biocompatibility (Thundimadathil, 2012). Therefore, the toxins present in the venom of *A. rondoniae* may be promising candidates to the investigation of therapeutic compounds.

The *in silico* anticancer and antimicrobial predictions have demonstrated to be important steps in our methodology, since it enabled a simple and fast screening of potential biological activities. It is important to highlight that the *in silico* predictions are indicatives of potential biological activities. However, the high scores in these predictions may not necessarily imply in real antimicrobial or anticancer activities. To confirm these hypotheses, experimental work should be performed in order to evaluate the biological activities of these peptides *in vitro* and *in vivo.* Although not definitive, the predictions suggest promising peptides and may serve as a guide in target selection for further steps of investigation, which is often a time-consuming task. These results demonstrate the effectiveness of a multiomics approach for toxin discovery, characterization and prospection of biological activities. The next steps would be the synthesis or expression of promising toxins to experimentally validate the activities observed *in silico*.

## Data Availability Statement

The datasets generated for this study can be found in the NCBI BioProject section under the accession code PRJNA633430 and BioSample SAMN14943686. The Transcriptome Shotgun Assembly was deposited at NCBI TSA under the accession GIOJ00000000. Mass spectrometry data were deposited to the ProteomeXchange Consortium via the PRIDE partner repository with the dataset identifier PXD019343.

## Author Contributions

GC and AT designed the concept of this study. MN, PS, IJ-D-A and AT designed experiments. GC, EK, and UO performed experiments. GC, MN and AT analyzed data. GC and AT prepared the draft and final version of the manuscript. All authors contributed to the article and approved the submitted version.

## Funding

This research was funded by Fundação de Amparo à Pesquisa do Estado de São Paulo (grants 2017/23771-3 to GC, 2013/07467-1 to PS and IJ-D-A, 2016/03839-0 and 2017/20106-9 to AT), Financiadora de Estudos e Projetos (FINEP) and Coordenação de Aperfeiçoamento de Pessoal de Nível Superior - Brasil (CAPES) - Finance Code 001.

## Conflict of Interest

The authors declare that the research was conducted in the absence of any commercial or financial relationships that could be construed as a potential conflict of interest.

## References

[B1] Abreu T. F. Sumitomo B. N. Nishiyama M. Y. Oliveira U. C. Souza G. H. M. F. Kitano E. S. (2017). Peptidomics of Acanthoscurria gomesiana spider venom reveals new toxins with potential antimicrobial activity. J. Proteomics 151, 232–242. 10.1016/j.jprot.2016.07.012 27436114

[B2] Altschul S. (1997). Gapped BLAST and PSI-BLAST: a new generation of protein database search programs. Nucleic Acids Res. 25, 3389–3402. 10.1093/nar/25.17.3389 9254694 PMC146917

[B3] Aronesty E. (2013). Comparison of Sequencing Utility Programs. Open Bioinforma. J. 7, 1–8. 10.2174/1875036201307010001

[B4] Arouri A. Dathe M. Blume A. (2009). Peptide induced demixing in PG/PE lipid mixtures: A mechanism for the specificity of antimicrobial peptides towards bacterial membranes? Biochim. Biophys. Acta Biomembr. 1788, 650–659. 10.1016/j.bbamem.2008.11.022 19118516

[B5] Bateman A. (2004). The Pfam protein families database. Nucleic Acids Res. 32, 138D–1141. 10.1093/nar/gkh121 PMC308855 14681378

[B6] Borges M. H. Figueiredo S. G. Leprevost F. V. De Lima M. E. Cordeiro M. do N. (2016). Venomous extract protein profile of Brazilian tarantula Grammostola iheringi: searching for potential biotechnological applications. J. Proteomics 136, 35–47. 10.1016/j.jprot.2016.01.013 26828374

[B7] Corzo G. Diego-García E. Clement H. Peigneur S. Odell G. Tytgat J. (2008). An insecticidal peptide from the theraposid Brachypelma smithi spider venom reveals common molecular features among spider species from different genera. Peptides 29, 1901–1908. 10.1016/j.peptides.2008.07.003 18687374

[B8] Deng M. Luo X. Xiao Y. Sun Z. Jiang L. Liu Z. (2014). Huwentoxin-XVI, an analgesic, highly reversible mammalian N-type calcium channel antagonist from Chinese tarantula Ornithoctonus huwena. Neuropharmacology 79, 657–667. 10.1016/j.neuropharm.2014.01.017 24467846

[B9] Duan Z. Cao R. Jiang L. Liang S. (2013). A combined de novo protein sequencing and cDNA library approach to the venomic analysis of Chinese spider Araneus ventricosus. J. Proteomics 78, 416–427. 10.1016/j.jprot.2012.10.011 23088928

[B10] Edwards I. A. Elliott A. G. Kavanagh A. M. Zuegg J. Blaskovich M. A. T. T. Cooper M. A. (2016). Contribution of Amphipathicity and Hydrophobicity to the Antimicrobial Activity and Cytotoxicity of β-Hairpin Peptides. ACS Infect. Dis. 2, 442–450. 10.1021/acsinfecdis.6b00045 27331141 PMC4906375

[B11] Escoubas P. King G. F. (2009). Venomics as a drug discovery platform. Expert Rev. Proteomics 6, 221–224. 10.1586/epr.09.45 19489692

[B12] Escoubas P. Rash L. (2004). Tarantulas: Eight-legged pharmacists and combinatorial chemists. Toxicon 43, 555–574. 10.1016/j.toxicon.2004.02.007 15066413

[B13] Felício M. R. Silva O. N. Gonçalves S. Santos N. C. Franco O. L. (2017). Peptides with Dual Antimicrobial and Anticancer Activities. Front. Chem. 5, 5. 10.3389/fchem.2017.00005 28271058 PMC5318463

[B14] Freitas-de-sousa L. A. Amazonas D. R. Sousa L. F. Sant’Anna S. S. Nishiyama J. M. Y. Serrano S. M. T. (2015). Comparison of venoms from wild and long-term captive Bothrops atrox snakes and characterization of Batroxrhagin, the predominant class PIII metalloproteinase from the venom of this species. Biochimie 118, 60–70. 10.1016/j.biochi.2015.08.006 26276061

[B15] Fry B. G. Roelants K. Champagne D. E. Scheib H. Tyndall J. D. A. King G. F. (2009). The Toxicogenomic Multiverse: Convergent Recruitment of Proteins Into Animal Venoms. Annu. Rev. Genomics Hum. Genet. 10, 483–511. 10.1146/annurev.genom.9.081307.164356 19640225

[B16] Fuertes G. Giménez D. Esteban-Martín S. Sánchez-Muñoz O. L. Salgado J. (2011). A lipocentric view of peptide-induced pores. Eur. Biophys. J. 40, 399–415. 10.1007/s00249-011-0693-4 21442255 PMC3070086

[B17] Gaspar D. Veiga A. S. Castanho M. A. R. B. (2013). From antimicrobial to anticancer peptides. A review. Front. Microbiol. 4, 294. 10.3389/fmicb.2013.00294 24101917 PMC3787199

[B18] Gaspar D. Freire J. M. Pacheco T. R. Barata J. T. Castanho M. A. R. B. (2015). Apoptotic human neutrophil peptide-1 anti-tumor activity revealed by cellular biomechanics. Biochim. Biophys. Acta Mol. Cell Res. 1853, 308–316. 10.1016/j.bbamcr.2014.11.006 25447543

[B19] Grabherr M. G. Haas B. J. Yassour M. Levin J. Z. Thompson D. A. Amit I. (2011). Full-length transcriptome assembly from RNA-Seq data without a reference genome. Nat. Biotechnol. 29, 644–652. 10.1038/nbt.1883 21572440 PMC3571712

[B20] Huang Y.-B. Wang X.-F. Wang H.-Y. Liu Y. Chen Y. (2011). Studies on Mechanism of Action of Anticancer Peptides by Modulation of Hydrophobicity Within a Defined Structural Framework. Mol. Cancer Ther. 10, 416–426. 10.1158/1535-7163.MCT-10-0811 21252288

[B21] Ikonomopoulou M. P. Fernandez-Rojo M. A. Pineda S. S. Cabezas-Sainz P. Winnen B. Morales R. A. V. (2018). Gomesin inhibits melanoma growth by manipulating key signaling cascades that control cell death and proliferation. Sci. Rep. 8, 11519. 10.1038/s41598-018-29826-4 30068931 PMC6070509

[B22] Jiang Z. Vasil A. II Hale J. D. Hancock R. E. W. Vasil M. L. Hodges R. S. (2008). Effects of net charge and the number of positively charged residues on the biological activity of amphipathic α-helical cationic antimicrobial peptides. Biopolymers 90, 369–383. 10.1002/bip.20911 18098173 PMC2761230

[B23] Johnson L. S. Eddy S. R. Portugaly E. (2010). Hidden Markov model speed heuristic and iterative HMM search procedure. BMC Bioinf. 11, 431. 10.1186/1471-2105-11-431 PMC2931519 20718988

[B24] Jung H. J. Kim P. Lee S. K. Lee C. W. Eu Y.-J. Lee D. G. (2006). Lipid membrane interaction and antimicrobial activity of GsMTx-4, an inhibitor of mechanosensitive channel. Biochem. Biophys. Res. Commun. 340, 633–638. 10.1016/j.bbrc.2005.12.046 16376854

[B25] Kaas Q. Craik D. J. (2015). Bioinformatics-Aided Venomics. Toxins (Basel) 7, 2159–2187. 10.3390/toxins7062159 26110505 PMC4488696

[B26] King G. F. Hardy M. C. (2013). Spider-Venom Peptides: Structure, Pharmacology, and Potential for Control of Insect Pests. Annu. Rev. Entomol. 58, 475–496. 10.1146/annurev-ento-120811-153650 23020618

[B27] King G. F. Gentz M. C. Escoubas P. Nicholson G. M. (2008). A rational nomenclature for naming peptide toxins from spiders and other venomous animals. Toxicon 52, 264–276. 10.1016/j.toxicon.2008.05.020 18619481

[B28] Kubista H. Mafra R. A. Chong Y. Nicholson G. M. Beirão P. S. L. Cruz J. S. (2007). CSTX-1, a toxin from the venom of the hunting spider Cupiennius salei, is a selective blocker of L-type calcium channels in mammalian neurons. Neuropharmacology 52, 1650–1662. 10.1016/j.neuropharm.2007.03.012 17517422

[B29] Kuhn-Nentwig L. Stöcklin R. Nentwig W. (2011). “Venom composition and strategies in spiders: is everything possible?,” in Advances in Insect Physiology (London: Academic Press), 1–86. 10.1016/B978-0-12-387668-3.00001-5

[B30] Kuhn-Nentwig L. Langenegger N. Heller M. Koua D. Nentwig W. (2019). The Dual Prey-Inactivation Strategy of Spiders—In-Depth Venomic Analysis of Cupiennius salei. Toxins (Basel) 11, 167. 10.3390/toxins11030167 PMC6468893 30893800

[B31] Langenegger N. Nentwig W. Kuhn-Nentwig L. (2019). Spider venom: Components, modes of action, and novel strategies in transcriptomic and proteomic analyses. Toxins (Basel) 11, 611. 10.3390/toxins11100611 PMC6832493 31652611

[B32] Langmead B. Salzberg S. L. (2012). Fast gapped-read alignment with Bowtie 2. Nat. Methods 9, 357–359. 10.1038/nmeth.1923 22388286 PMC3322381

[B33] Lau C. H. Y. King G. F. Mobli M. (2016). Molecular basis of the interaction between gating modifier spider toxins and the voltage sensor of voltage-gated ion channels. Sci. Rep. 6, 34333. 10.1038/srep34333 27677715 PMC5039624

[B34] Lee S.-Y. MacKinnon R. (2004). A membrane-access mechanism of ion channel inhibition by voltage sensor toxins from spider venom. Nature 430, 232–235. 10.1038/nature02632 15241419

[B35] Li Q. Zhao Z. Zhou D. Chen Y. Hong W. Cao L. (2011). Virucidal activity of a scorpion venom peptide variant mucroporin-M1 against measles, SARS-CoV and influenza H5N1 viruses. Peptides 32, 1518–1525. 10.1016/j.peptides.2011.05.015 21620914 PMC7115635

[B36] Liao Z. Yuan C. Deng M. Li J. Chen J. Yang Y. (2006). Solution structure and functional characterization of jingzhaotoxin-XI: a novel gating modifier of both potassium and sodium channels. Biochemistry 45, 15591–15600. 10.1021/bi061457+ 17176080

[B37] Lomazi R. L. Nishiduka E. S. Silva P. II Tashima A. K. (2018). “Identification of Peptides in Spider Venom Using Mass Spectrometry,” in Peptidomics - Methods and Strategies. Eds. Fricker L. D. Schrader M. (New York: Springer), 359–367. 10.1007/978-1-4939-7537-2_24 29476524

[B38] Lucas S. M. Da Silva P. II Bertani R. Costa Cardoso J. L. (1994). Mygalomorph spider bites: A report on 91 cases in the State of São Paulo, Brazil. Toxicon 32, 1211–1215. 10.1016/0041-0101(94)90350-6 7846691

[B39] Manavalan B. Basith S. Shin T. H. Choi S. Kim M. O. Lee G. (2017). MLACP: machine-learning-based prediction of anticancer peptides. Oncotarget 8, 77121–77136. 10.18632/oncotarget.20365 29100375 PMC5652333

[B40] Mebs D. (2001). Toxicity in animals. Trends in evolution? Toxicon 39, 87–96. 10.1016/S0041-0101(00)00155-0 10936625

[B41] Meher P. K. Sahu T. K. Saini V. Rao A. R. (2017). Predicting antimicrobial peptides with improved accuracy by incorporating the compositional, physico-chemical and structural features into Chou’s general PseAAC. Sci. Rep. 7, 42362. 10.1038/srep42362 28205576 PMC5304217

[B42] Mobli M. Undheim E. A. B. Rash L. D. (2017). “Modulation of Ion Channels by Cysteine-Rich Peptides,” in Ion Channels DownUnder (Cambridge: Elsevier Inc.), 199–223. 10.1016/bs.apha.2017.03.001 28528669

[B43] Mourão C. B. F. Heghinian M. D. Barbosa E. A. Marí F. Bloch C. Restano-Cassulini R. (2013). Characterization of a Novel Peptide Toxin from Acanthoscurria paulensis Spider Venom: A Distinct Cysteine Assignment to the HWTX-II Family. Biochemistry 52, 2440–2452. 10.1021/bi4000035 23496776

[B44] Osorio D. Rondon-Villarreal P. Torres R. (2015). Peptides: A Package for Data Mining of Antimicrobial Peptides. R. J. 7, 4–14. 10.32614/RJ-2015-001

[B45] Osteen J. D. Herzig V. Gilchrist J. Emrick J. J. Zhang C. Wang X. (2016). Selective spider toxins reveal a role for the Nav1.1 channel in mechanical pain. Nature 534, 494–499. 10.1038/nature17976 27281198 PMC4919188

[B46] Pérez-Peinado C. Defaus S. Andreu D. (2020). Hitchhiking with Nature: Snake Venom Peptides to Fight Cancer and Superbugs. Toxins (Basel) 12, 255. 10.3390/toxins12040255 PMC7232197 32326531

[B47] Perez-Riverol Y. Csordas A. Bai J. Bernal-Llinares M. Hewapathirana S. Kundu D. J. (2019). The PRIDE database and related tools and resources in 2019: improving support for quantification data. Nucleic Acids Res 47, D442–D450. 10.1093/nar/gky1106 30395289 PMC6323896

[B48] Paredes-Gamero E. J. Casaes-Rodrigues R. L. Moura G. E. D. D. Domingues T. M. Buri M. V. Ferreira V. H. C. (2012). Cell-Permeable Gomesin Peptide Promotes Cell Death by Intracellular Ca 2+ Overload. Mol. Pharm. 9, 2686–2697. 10.1021/mp300251j 22873645

[B49] Pedroso A. P. Souza A. P. Dornellas A. P. S. Oyama L. M. Nascimento C. M. O. Santos G. M. S. (2017). Intrauterine Growth Restriction Programs the Hypothalamus of Adult Male Rats: Integrated Analysis of Proteomic and Metabolomic Data. J. Proteome Res. 16, 1515–1525. 10.1021/acs.jproteome.6b00923 28314371

[B50] Pineda S. S. Chaumeil P.-A. Kunert A. Kaas Q. Thang M. W. C. C. Le L. (2018). ArachnoServer 3.0: an online resource for automated discovery, analysis and annotation of spider toxins. Bioinformatics 34, 1074–1076. 10.1093/bioinformatics/btx661 29069336

[B51] Rappsilber J. Mann M. Ishihama Y. (2007). Protocol for micro-purification, enrichment, pre-fractionation and storage of peptides for proteomics using StageTips. Nat. Protoc. 2, 1896–1906. 10.1038/nprot.2007.261 17703201

[B52] Rates B. Prates M. V. Verano-Braga T. da Rocha Â. P. Roepstorff P. Borges C. L. (2013). μ-Theraphotoxin-An1a: Primary structure determination and assessment of the pharmacological activity of a promiscuous anti-insect toxin from the venom of the tarantula Acanthoscurria natalensis (Mygalomorphae, Theraphosidae). Toxicon 70, 123–134. 10.1016/j.toxicon.2013.04.013 23651762

[B53] Riciluca K. C. T. Sayegh R. S. R. Melo R. L. Silva P. II (2012). Rondonin an antifungal peptide from spider (Acanthoscurria rondoniae) haemolymph. Results Immunol. 2, 66–71. 10.1016/j.rinim.2012.03.001 24371568 PMC3862377

[B54] Rocha-e-Silva T. A. A. Sutti R. Hyslop S. (2009). Milking and partial characterization of venom from the Brazilian spider Vitalius dubius (Theraphosidae). Toxicon 53, 153–161. 10.1016/j.toxicon.2008.10.026 19032960

[B55] Rozek T. Wegener K. L. Bowie J. H. Olver I. N. Carver J. A. Wallace J. C. (2000). The antibiotic and anticancer active aurein peptides from the Australian Bell Frogs Litoria aurea and Litoria raniformis. Eur. J. Biochem. 267, 5330–5341. 10.1046/j.1432-1327.2000.01536.x 10951191

[B56] Ruta V. Jiang Y. Lee A. Chen J. MacKinnon R. (2003). Functional analysis of an archaebacterial voltage-dependent K+ channel. Nature 422, 180–185. 10.1038/nature01473 12629550

[B57] Saez N. J. Senff S. Jensen J. E. Er S. Y. Herzig V. Rash L. D. (2010). Spider-venom peptides as therapeutics. Toxins (Basel) 2, 2851–2871. 10.3390/toxins2122851 22069579 PMC3153181

[B58] Sanggaard K. W. Bechsgaard J. S. Fang X. Duan J. Dyrlund T. F. Gupta V. (2014). Spider genomes provide insight into composition and evolution of venom and silk. Nat. Commun. 5, 3765. 10.1038/ncomms4765 24801114 PMC4273655

[B59] Schendel V. Rash L. D. Jenner R. A. Undheim E. A. B. (2019). The Diversity of Venom: The Importance of Behavior and Venom System Morphology in Understanding Its Ecology and Evolution. Toxins (Basel) 11, 666. 10.3390/toxins11110666 PMC6891279 31739590

[B60] Seo M.-D. Won H.-S. Kim J.-H. Mishig-Ochir T. Lee B.-J. (2012). Antimicrobial Peptides for Therapeutic Applications: A Review. Molecules 17, 12276–12286. 10.3390/molecules171012276 23079498 PMC6268056

[B61] Silva P. II Daffre S. Bulet P. (2000). Isolation and characterization of gomesin, an 18-residue cysteine-rich defense peptide from the spider Acanthoscurria gomesiana hemocytes with sequence similarities to horseshoe crab antimicrobial peptides of the tachyplesin family. J. Biol. Chem. 275, 33464–33470. 10.1074/jbc.M001491200 10942757

[B62] Silva J. C. Gorenstein M. V. Li G.-Z. Vissers J. P. C. Geromanos S. J. (2006). Absolute Quantification of Proteins by LCMS E. Mol. Cell. Proteomics 5, 144–156. 10.1074/mcp.M500230-MCP200 16219938

[B63] Thundimadathil J. (2012). Cancer Treatment Using Peptides: Current Therapies and Future Prospects. J. Amino Acids 2012, 1–13. 10.1155/2012/967347 PMC3539351 23316341

[B64] Undheim E. A. B. Grimm L. L. Low C.-F. Morgenstern D. Herzig V. Zobel-Thropp P. (2015). Weaponization of a Hormone: Convergent Recruitment of Hyperglycemic Hormone into the Venom of Arthropod Predators. Structure 23, 1–10. 10.1016/j.str.2015.05.003 26073605

[B65] Vetter R. S. Isbister G. K. (2008). Medical Aspects of Spider Bites. Annu. Rev. Entomol. 53, 409–429. 10.1146/annurev.ento.53.103106.093503 17877450

[B66] Vilas Boas L. C. P. Campos M. L. Berlanda R. L. A. de Carvalho Neves N. Franco O. L. (2019). Antiviral peptides as promising therapeutic drugs. Cell. Mol. Life Sci. 76, 3525–3542. 10.1007/s00018-019-03138-w 31101936 PMC7079787

[B67] Wachinger M. Kleinschmidt A. Winder D. Von Pechmann N. Ludvigsen A. Neumann M. (1998). Antimicrobial peptides melittin and cecropin inhibit replication of human immunodeficiency virus 1 by suppressing viral gene expression. J. Gen. Virol. 79, 731–740. 10.1099/0022-1317-79-4-731 9568968

[B68] Windley M. J. Herzig V. Dziemborowicz S. A. Hardy M. C. King G. F. Nicholson G. M. (2012). Spider-Venom Peptides as Bioinsecticides. Toxins (Basel) 4, 191–227. 10.3390/toxins4030191 22741062 PMC3381931

[B69] Zelanis A. Keiji Tashima A. (2014). Unraveling snake venom complexity with ‘omics’ approaches: Challenges and perspectives. Toxicon 87, 131–134. 10.1016/j.toxicon.2014.05.011 24878375

[B70] Zhang J. Xin L. Shan B. Chen W. Xie M. Yuen D. (2012). PEAKS DB: De Novo Sequencing Assisted Database Search for Sensitive and Accurate Peptide Identification. Mol. Cell. Proteomics 11, M111.010587. 10.1074/mcp.M111.010587 PMC3322562 22186715

[B71] Zhang C. Yang M. Ericsson A. C. (2019). Antimicrobial Peptides: Potential Application in Liver Cancer. Front. Microbiol. 10, 1257. 10.3389/fmicb.2019.01257 31231341 PMC6560174

[B72] Zhao H. Zhou J. Zhang K. Chu H. Liu D. Poon V. K.-M. (2016). A novel peptide with potent and broad-spectrum antiviral activities against multiple respiratory viruses. Sci. Rep. 6, 22008. 10.1038/srep22008 26911565 PMC4766503

[B73] Zhou X. Ma T. Yang L. Peng S. Li L. Wang Z. (2020). Spider venom-derived peptide induces hyperalgesia in Nav1.7 knockout mice by activating Nav1.9 channels. Nat. Commun. 11, 2293. 10.1038/s41467-020-16210-y 32385249 PMC7210961

[B74] Zobel-Thropp P. A. Bulger E. A. Cordes M. H. J. Binford G. J. Gillespie R. G. Brewer M. S. (2018). Sexually dimorphic venom proteins in long-jawed orb-weaving spiders ( Tetragnatha ) comprise novel gene families. PeerJ 6, e4691. 10.7717/peerj.4691 29876146 PMC5985773

